# Mesenchymal Stem Cells in Clinical Trials for Immune Disorders

**DOI:** 10.1055/s-0044-1788044

**Published:** 2024-06-27

**Authors:** Zongjin Li, Zhibo Han, Zhong-Chao Han

**Affiliations:** 1 Department of Pathophysiology, Nankai University School of Medicine, Tianjin, China; 2 Academy of Medical Engineering and Translational Medicine, Tianjin University, Tianjin, China; 3 Beijing Engineering Laboratory of Perinatal Stem Cells, Beijing Institute of Health and Stem Cells, Health & Biotech Co., Beijing, China

Mesenchymal stem cells (MSCs) are a population of adult stem cells found in various tissues, including bone marrow, adipose tissue, and perinatal-related tissues. 1 MSCs have emerged as a promising therapeutic approach due to their unique properties, including regeneration and immunomodulation. The therapeutic mechanism of MSCs is complex and not fully understood, but they are believed to involve several different processes. One important mechanism is the paracrine secretion of growth factors, cytokines, and other signaling molecules. 2 3 These molecules can help to promote tissue repair, reduce inflammation, and modulate the immune system by interacting with various components of tissue microenvironments and modulating immune responses. 4 The immunomodulatory effects of MSCs are one of their most intriguing and therapeutically valuable properties. 5

**Fig. 1:**
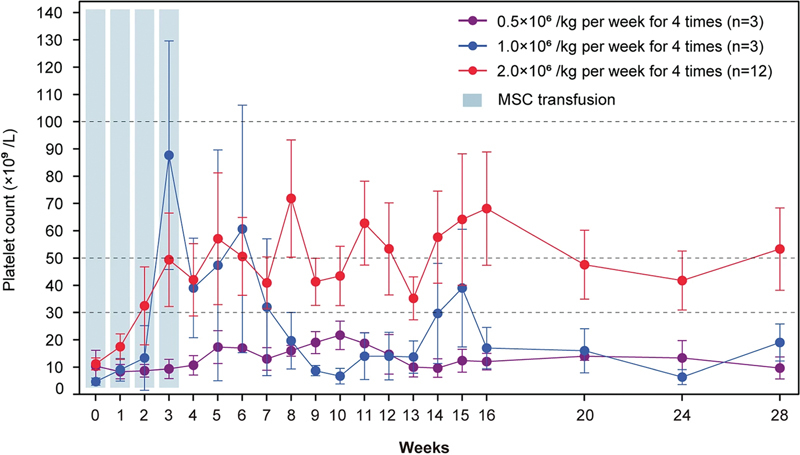
Platelet counts over time in all patients. Platelet counts of all enrolled patients from baseline to 24 weeks after the completion of UC-MSC administration. Reproduced with permission from Chen et al 2024. 6 UC-MSC, umbilical cord-derived mesenchymal stem cell.

A recent prospective, single-arm, phase I trial described the efficacy and safety of human umbilical cord-derived MSCs (UC-MSCs) in the treatment of refractory immune thrombocytopenia (ITP), highlighting the effectiveness and good tolerance of UC-MSCs in treating refractory ITP. 6 These findings offer insights that may advance our understanding of the clinical application of MSCs in immune diseases. This trial revealed that the overall response rate was 44.4% (8/18) among all enrolled patients with mild adverse events. All patients in the 2.0 × 10 ^6^ cells/kg group achieved a platelet count of approximately 50 × 10 ^9^ /L for up to 28 weeks ( Fig. 1 ), which revealed a dose-dependent trend of platelet count. Most importantly, bleeding symptoms can be completely relieved in 60.0 to 75.0% of patients after treatment, and no serious treatment-related emergent adverse events occur.

ITP is an acquired autoimmune hemorrhagic disease resulting from an imbalance of immune tolerance. Refractory ITP refers to patients who have failed multiple therapies, and current guidelines have limited effectiveness in alleviating bleeding symptoms. 6 In this clinical trial, off-the-shelf GMP (Good Manufacturing Practice)-grade UC-MSCs from Tianjin Amcellgene Co., Ltd., were used. 7 8 MSCs are also known to be heterogeneous populations with variable functions. Many studies have comparatively analyzed the differential properties and biological functions of MSCs derived from perinatal and adult tissues, including their molecular profiles, differentiation potentials, proliferation/clonogenic formation capacities, immunomodulatory functions, and hematopoietic support abilities. MSCs derived from perinatal tissues such as the umbilical cord and placenta have attracted attention because of their noninvasive isolation methods and minimal ethical issues. Moreover, compared with adult tissue-derived MSCs, perinatal tissue-derived MSCs are young cells without increased possibilities of mutation. 1 5 The banking of UC-MSCs represents a noninvasive, simple, and safe means for harvesting MSCs. 9 In this phase I trial, systemic infusion of UC-MSCs was applied, and the circulation dynamics of UC-MSCs were detected by the *SRY* gene in peripheral blood. However, UC-MSCs were not detectable in the blood 8 hours after injection. The therapeutic mechanisms of UC-MSCs were investigated, and the data revealed a temporary decrease in T-cell percentages during the infusion of UC-MSCs, followed by a gradual increase in the proportion of CD8 + CD28− suppressive T-cell subsets.

**Fig. 2:**
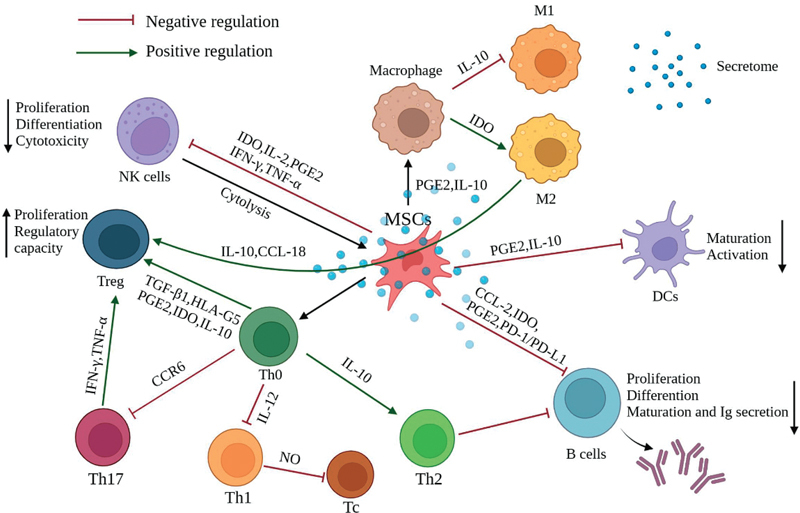
MSCs act as immunomodulators, influencing the behavior of other immune cells to create a more balanced immune response. This property makes them a potential treatment for a wide range of diseases characterized by an imbalanced immune response, such as autoimmune disorders, graft-versus-host disease, and inflammatory conditions. Reproduced with permission from Huang et al 2022. 12 MSC, mesenchymal stem cell.

MSCs interact with various components of the immune system and modulate immune responses in several ways. First, MSCs can directly interact with T cells through cell surface molecules such as PD-L1, which binds to the PD-1 receptor on T cells, leading to reduced T-cell proliferation and activation. 10 Secondly, MSCs secrete various soluble factors, such as prostaglandin E2 (PGE2), indoleamine 2,3-dioxygenase (IDO), transforming growth factor-beta (TGF-β), and hepatocyte growth factor, all of which contribute to the suppression of T-cell proliferation and activity. 11 In addition, MSCs inhibit B-cell maturation through the release of soluble factors such as PGE2 and by producing IDO, decreasing the differentiation of B cells into plasma cells and reducing antibody production. 12 However, opposite results were observed in which MSCs promoted the proliferation and differentiation of B cells. 2 Furthermore, MSCs reduce the cytotoxic activity of natural killer (NK) cells and their ability to produce proinflammatory cytokines. MSCs influence macrophages to adopt an anti-inflammatory M2 phenotype rather than a proinflammatory M1 phenotype mediated by MSC-derived IL-10, TGF-β, and PGE2. 13 The immunomodulatory capacity of MSCs is a critical aspect of their therapeutic potential, enabling them to create a more favorable environment for tissue repair and regeneration by modulating immune responses in a controlled manner ( Fig. 2 ). The immunomodulatory properties of MSCs have significant implications for treating various inflammatory and autoimmune conditions, such as graft-versus-host disease, rheumatoid arthritis, multiple sclerosis, systemic lupus erythematosus, Crohn's disease, and ulcerative colitis. 12

**Fig. 3:**
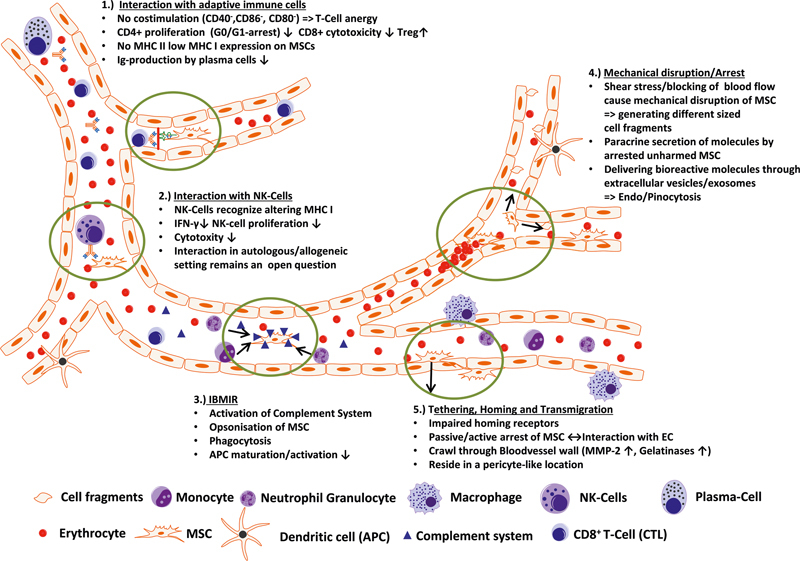
Interactions of MSCs with tissue microenvironments after intravenous administration. Reproduced with permission from Leibacher and Henschler 2016. 16 MSC, mesenchymal stem cell.

For MSC therapy, intravenous infusions of MSCs are a prominent priority for repeated injection, noninvasive procedures, and easy operation. The intravenously transplanted MSCs disappeared in a short time, and an increase in the number of MSCs might not prolong cell retention. MSCs might interact within the blood circulation after intravenous administration to function in immunomodulation ( Fig. 3 ). MSCs can modulate the immune response by interacting with adaptive immune cells and NK cells and exerting paracrine effects. However, most MSCs return to and reach the lung after intravenous administration. 14 Therefore, how intravenously infused MSCs bridge the spatial gap between the lung and other organs to achieve immunomodulatory effects remains a challenge. Recently, the process by which exogenous cells in an organ affect distant recipient cells in another organ has been recognized as transorgan communication. 15 A recent study demonstrated that intravenous infusion of MSCs could secrete extracellular vesicles (EVs) and contribute to the therapeutic effects of MSCs. 14 This study revealed that intravenously transplanted MSCs could serve as an endocrine reservoir to secrete EVs into the blood continuously and gradually to promote transorgan communication.

In summary, this phase I trial showed that UC-MSCs can increase platelet counts and improve bleeding symptoms in refractory ITP patients. MSCs have immense potential for treating various diseases because of their immunomodulatory properties. Research to address the distribution and metabolism of MSCs in organ tissues is vital to translate this potential into effective and widely available clinical applications. As our understanding of MSCs and their interactions with the immune system deepens, we can look forward to a future where MSC therapy becomes a mainstay in treating a broad spectrum of immune-related conditions, including refractory ITP.
